# Quantifying the fall in mortality associated with interventions related to hypertensive diseases of pregnancy

**DOI:** 10.1186/1471-2458-11-S3-S8

**Published:** 2011-04-13

**Authors:** Carine Ronsmans, Oona Campbell

**Affiliations:** 1 Infectious Disease Epidemiology Unit, London School of Hygiene and Tropical Medicine, London, UK

## Abstract

**Background:**

In this paper we review the evidence of the effect of health interventions on mortality reduction from hypertensive diseases in pregnancy (HDP). We chose HDP because they represent a major cause of death in low income countries and evidence of effect on maternal mortality from randomised studies is available for some interventions.

**Methods:**

We used four approaches to review the evidence of the effect of interventions to prevent or treat HDP on mortality reduction from HDP. We first reviewed the Cochrane Library to identify systematic reviews and individual trials of the efficacy of single interventions for the prevention or treatment of HDP. We then searched the literature for articles quantifying the impact of maternal health interventions on the reduction of maternal mortality at the population level and describe the approaches used by various authors for interventions related to HDP. Third, we examined levels of HDP-specific mortality over time or between regions in an attempt to quantify the actual or potential reduction in mortality from HDP in these regions or over time. Lastly, we compared case fatality rates in women with HDP-related severe acute maternal morbidity with those reported historically in high income countries before any effective treatment was available.

**Results:**

The Cochrane review identified 5 effective interventions: routine calcium supplementation in pregnancy, antiplatelet agents during pregnancy in women at risk of pre-eclampsia, Magnesium sulphate (MgS04) for the treatment of eclampsia, MgS04 for the treatment of pre-eclampsia, and hypertensive drugs for the treatment of mild to moderate hypertension in pregnancy.

We found 10 studies quantifying the effect of maternal health interventions on reducing maternal mortality from HDP, but the heterogeneity in the methods make it difficult to draw uniform conclusions for effectiveness of interventions at various levels of the health system. Most authors include a health systems dimension aimed at separating interventions that can be delivered at the primary or health centre level from those that require hospital treatment, but definitions are rarely provided and there is no consistency in the types of interventions that are deemed effective at the various levels.

The low levels of HDP related mortality in rural China and Sri Lanka suggest that reductions of 85% or more are within reach, provided that most women give birth with a health professional who can refer them to higher levels of care when necessary. Results from studies of severe acute maternal morbidity in Indonesia and Bolivia also suggest that mortality in women with severe pre-eclampsia or eclampsia in hospital can be reduced by more than 84%, even when the women arrive late.

**Conclusions:**

The increasing emphasis on the rating of the quality of evidence has led to greater reliance on evidence from randomised controlled trials to estimate the effect of interventions. Yet evidence from randomised studies is often not available, the effects observed on morbidity may not translate in to mortality, and the distinction between efficacy and effectiveness may be difficult to make. We suggest that more use should be made of observational evidence, particularly since such data represent the actual effectiveness of packages of interventions in various settings.

## Introduction

The fifth Millennium Development Goal has set targets for the reduction of maternal mortality by 2015, but progress has been slow [1,2]. Effective interventions to reduce maternal deaths exist but they are often not available to women in poor countries [3]. Where resources are limited, information on the costs and health effects of interventions is thought to be important to aid decisions on how to reach the MDG-goals [4]. Such information may help to determine what can be achieved with existing resources, and to decide how additional funds can be used to maximise the chances of achieving the MDG-goals [4].

WHO estimates that 88 to 98 percent of maternal deaths are avoidable with moderate levels of health care [5]. This deceptively simple statement hides the complexities underlying the assessment of the health effects of interventions [6]. First, evidence of the efficacy of interventions needs to be available. In maternal health, reliable evidence of an effect on maternal mortality is rarely available and reliance on lower quality evidence – by current scientific standards - is often necessary [3]. Second, a distinction needs to be made between efficacy and effectiveness. Effectiveness, taking into account the coverage and quality of service delivery is thought to be more representative of the real world, but it requires robust evidence from a large range of programme settings. Third, interventions act on disease incidence, severity or mortality, and effect on one outcome does not easily translate into effect on the other. Lastly, interventions are generally delivered in packages, and assessments of health effects need to take account of interactions in the effect between interventions. Information on the joint effect of multiple interventions is rarely available from trials, and assumptions on how they interact need to be made.

In this paper we review the evidence of the effect of health interventions on mortality reduction from hypertensive diseases in pregnancy (HDP). We chose HDP because they represent a major cause of death in low income countries [7] and evidence of effect on maternal mortality from randomised studies is available for some interventions [3]. In our review we first summarise efficacy data from randomised controlled trials, separating effects on mortality from those on morbidity. We then review the literature on population models quantifying the effect of HDP-related maternal health interventions on mortality and examine the methods used. Lastly, we explore alternative methods to determine the effectiveness of comprehensive packages of health interventions on mortality by examining differentials in HDP-related mortality and case fatality over time and between countries or regions.

## Methods

We used four approaches to review the evidence of the effect of interventions to prevent or treat HDP on mortality reduction from HDP. We first reviewed the Cochrane Library to identify systematic reviews and individual trials of the efficacy of single interventions for the prevention or treatment of HDP. We then searched the literature for articles quantifying the impact of maternal health interventions on the reduction of maternal mortality at the population level and describe the approaches used by various authors for interventions related to HDP. Third, we examined levels of HDP-specific mortality over time or between regions in an attempt to quantify the actual or potential reduction in mortality from HDP in these regions or over time. Lastly, we compared case fatality rates in women with HDP-related severe acute maternal morbidity with those reported historically in high income countries before any effective treatment was available.

### Cochrane reviews of the efficacy of the prevention or treatment of HDP on maternal health outcomes

The Cochrane database of Systematic Reviews (June 2010) was searched using the terms hypertension during pregnancy, pre-eclampsia and eclampsia. Reviews were included if they reported the effect of an intervention on any of the following HDP-related maternal outcomes: maternal or HDP-related death, recurring convulsions, eclampsia, pre-eclampsia, placental abruption, intra- or postpartum bleeding, and hypertension. Studies from all countries were considered because few or no data are available from low-income countries. Reviews with no evidence of effect on any of the HDP-related maternal outcomes (i.e. confidence intervals on the RR crossed the no-effect line) were excluded. Reviews with evidence of effect on at least one of the HDP-related maternal outcomes but with less than 50 outcome events were also excluded. We also searched the Cochrane Central Register of Controlled Trials (June 2010) for trials published from 2000 onwards using the same search criteria as above. We selected single interventions for which no Cochrane review had been performed by June 2010. We followed the standard recommended steps for data extraction, summary of the evidence and effect estimates [8].

### Population models quantifying the impact of the prevention or treatment of HDP on mortality from HDP at the population level

We searched PubMed for articles quantifying the impact of maternal health interventions on the reduction of maternal mortality at the population level, using the terms interventions, maternal mortality and effectiveness in our search. Reference lists from all relevant articles were checked. We included articles reporting the effect of preventive or curative interventions on HDP-related mortality, regardless of the definitions used. Information was extracted on interventions, the health systems level at which the intervention was delivered, the mortality outcome, the reported risk reduction on mortality outcomes, the methods for estimating effect sizes, and the references quoted.

### Comparing current and historic maternal mortality ratios across regions or countries

To obtain HDP-specific maternal mortality ratios by region we applied the proportion of deaths from HDP by region [^7^] to region-specific estimates of maternal mortality for 2005.[9] We also obtained data on the HDP-specific maternal mortality ratio over time in England & Wales between 1912 and 2005 [10,11], in Sweden between 1933 and 1975 [12], in Sri Lanka between 1933-200 [13] and in urban and rural China between 2000 and 2007 [14]. To estimate the actual or potential risk reduction in mortality from HDP we compared the levels of maternal mortality in various regions in 2005 with that in high income countries in 2005; and the earliest estimates of maternal mortality with the latest estimates. The potential risk reduction was computed as (earliest [or highest] maternal mortality ratio – lowest maternal mortality ratio) / earliest [or highest] maternal mortality ratio).

### Comparing current and historic estimates of fatality in women with SAMM related to HDP

Lastly, we used two recent reviews of severe acute maternal morbidity (SAMM) in high and low income countries [15,16] and a study from Sweden [12] to identify studies reporting on SAMM. Studies were selected if they reported the case fatality rate of HDP, defined as the number of deaths from HDP divided by the total number of SAMM from HDP. The potential risk reduction was computed as ((observed case fatality rate – natural case fatality rate) / observed case fatality rate). The natural case fatality rate from HDP was estimated from historical data in England & Wales (25% [10]) and Sweden (42% [12]).

## Results

### Cochrane reviews of the efficacy of the prevention or treatment of HDP on maternal health outcomes

Twenty nine reviews reported the effect of interventions on HDP-related health outcomes. Eighteen interventions (reported in 19 reviews) were excluded because there was no evidence of effect on any of the maternal outcomes listed. Excluded interventions were supplementation or prophylactic administration of: antoxidants [17], zinc [18], Vitamin C [19], Vitamin E [20], pyridoxine [21], oestrogen [22], progesterone [23], marine oil [24], garlic [25], diuretics [26], or nitric oxide [27] during pregnancy. Increased energy and protein intake [28], altered dietary salt (2 reviews) [29,30] and exercise [31] during pregnancy were also excluded. Bed rest for hypertension [32], low dose dopamine for women with severe pre-eclampsia [33], plasma volume expansion for treatment of pre-eclampsia [34] or early delivery for severe pre-eclampsia before 34 weeks gestation [35] were also not found to be associated with any of the HDP-related maternal outcomes.

Two further reviews had evidence of effect on at least one of the HDP-related maternal outcomes, but the number of events was insufficient to warrant inclusion. Women with severe hypertension allocated calcium channel blockers rather than hydralazine were less likely to have persistent high blood pressure (five trials, RR 0.33 95% CI 0.15-0.70), but the total number of women with persistent high blood pressure was only 31.[36] Rest and nutritional supplementation in women at moderate risk of eclampsia reduces the risk of pre-eclampsia (one trial, RR 0.13 95% CI 0.03-0.51) but only 18 women developed pre-eclampsia in the trial [37].

We read 251 abstracts reported in the Cochrane Central Register of Controlled Trials between 2000 and June 2010. Two interventions had evidence of effect on HDP-related outcomes. One trial assessed the effect of Coenzyme Q10 supplementation during pregnancy in women at increased risk of pre-eclampsia and found a 44% reduction in pre-eclampsia (RR0.56; 95% CI 0.33-0.96) [38]. Another trial compared induction of labour with expectant monitoring for gestational hypertension or mild pre-eclampsia after 36 weeks gestation [39]. No cases of death or eclampsia were recorded, but women in the intervention group had a 29% lower risk of a composite indicator of poor maternal outcome (RR 0.71, 95% CI 0.59-0.86). Poor maternal outcome was defined as maternal mortality, maternal morbidity (eclampsia, HELLP syndrome, pulmonary oedema, thromboembolic disease, or placental abruption), progression to severe disease (at least one measurement during ante-partum or post-partum period of systolic blood pressure ≥170 mm Hg, diastolic blood pressure ≥110 mm Hg, or proteinuria ≥5 g per 24 h), and major post-partum haemorrhage. These two interventions were not included because consistency of effect across study populations could not be assessed.

The results of included reviews are shown in Additional File 1. We report two prevention strategies: routine calcium supplementation in pregnancy [40] and antiplatelet agents during pregnancy in women at risk of pre-eclampsia [41] and three treatment interventions: Magnesium sulphate (MgS04) for the treatment of eclampsia (3 reviews) [42-44] MgS04 for the treatment of pre-eclampsia [45], and hypertensive drugs for the treatment of mild to moderate hypertension in pregnancy [46]. The review documenting the effect of oral beta-blockers for hypertension [47] is not reported separately because beta-blockers are included in a later review of hypertensive drugs [46]. All studies are randomized controlled trials, and the quality of the trials is generally high. The number of deaths reported was sufficient in only one review [42] and one review combined death with severe morbidity to increase the number of adverse events [40].

There is no doubt that treating women with eclampsia with MgS04 reduces the risk of maternal death compared to diazepam (RR 0.59 95% CI 0.37-0.94), though the effect against placebo is not known. MgS04 is also effective for the treatment of pre-eclampsia: treating women with pre-eclampsia with MgS04 reduces their risk of eclampsia (RR 0.41 95% CI 0.29-0.58) and placental abruption (RR 0.64 95% CI 0.50-0.83), though there is insufficient evidence to draw conclusions with regard to the risk of death. The efficacy of the treatment of hypertension in pregnancy is less clear. Antihypertensive drugs in women with mild to moderate hypertension do not lower the risk of pre-eclampsia and there are insufficient numbers of events to assess their effect on risk of eclampsia or maternal death. Antihypertensive drugs do halve the risk of developing severe hypertension (RR 0.50 95% CI 0.41-0.61) [46].

Routine calcium supplementation during pregnancy halves the risk of pre-eclampsia (RR 0.45 95% CI 0.31-0.65), and reduces the occurrence of a composite outcome of death or serious morbidity (RR 0.80 95% CI 0.65-0.97). The reduction in the risk of pre-eclampsia is greatest for women at high risk of pre-eclampsia (5 trials, 587 women: RR 0.22, 95% CI 0.12-0.42), and for those with low baseline calcium intake (8 trials, 10,678 women: RR 0.36, 95% CI 0.20-0.65). There is a 17% reduction in the risk of pre-eclampsia with the use of antiplatelet agents – mostly low dose aspirin - during pregnancy in women at risk of pre-eclampsia (RR 0.83 95% CI 0.77-0.89). However, there are no significant differences between antiplatelet agents and placebo in the risk of eclampsia or maternal death.

### Population models quantifying the impact of the prevention or treatment of HDP on mortality from HDP at the population level

We found 15 studies quantifying the effect of maternal health interventions on reducing maternal mortality at the population level, ten of which are included here (Additional File 2). Five studies were excluded because they did not report on HDP specifically [48-51] or because findings were only presented in a chart with no information on assumptions or the data underlying the chart [52].

The approach to classifying interventions varies greatly (Additional File 2). Most authors include a health systems dimension aimed at separating interventions that can be delivered at the primary or health centre level from those that require hospital treatment, though definitions are rarely provided and there is no consistency in the types of interventions that are deemed effective at the various levels. At the hospital level, effective interventions usually consist of MgS04 for the treatment of eclampsia [53-59], and later studies also include MgS04 for the treatment of pre-eclampsia.[55,58-60] Caesarean section is generally listed as part of the hospital package, and Graham et al (2006) [55] also include calcium supplementation, low dose aspirin, antioxidants and antihypertensive drugs as effective interventions at the hospital level.

Primary care interventions tend to focus on screening and supportive care, with a reliance on referral care for the management of women at high risk or with complications. Maine (1991) [61] separates referral to rural hospitals from referral to urban hospitals, arguing that rural hospitals are more accessible and therefore more effective. Four authors report primary care to be effective in the absence of referral to higher levels of care. Interventions include sedatives [61], calcium supplementation [62], or a comprehensive primary care package including the treatment of hypertension, routine calcium supplementation, low dose aspirin in high risk women and MgS04 for the treatment of pre-eclampsia.[60] Graham et al [54] report that risk screening and management at the first aid and basic obstetric care level has a substantial effect on reducing mortality from hypertensive diseases in the absence of referral care, though the specific interventions deemed to be effective in managing risk factors at primary care level are not listed.

HDP-related mortality outcomes range from deaths due to eclampsia to deaths from eclampsia and pre-eclampsia or hypertensive disorders of pregnancy. No definitions are provided.

The approach to estimating effect sizes includes reliance on evidence from expert opinion, previous models, a single trial or systematic reviews. Four reviews rely on expert opinion, but the authors do not specify the characteristics or the numbers of experts involved nor how the expert opinion is arrived at [55].[56][57][61] WHO (1994) [53] uses one of Maine’s early expert opinion-based estimates to calculate the effect of the comprehensive Mother Baby Package on mortality reductions from eclampsia. Interestingly, WHO relies on the effect estimate for the strategy involving health centres with referral to rural hospitals rather than the slightly more conservative estimate for a strategy involving health centres with referral to urban hospitals. In later studies, Prata [56] and Graham [55] also use WHO’s effect estimate as the main source for their effect sizes.

Seven studies rely on evidence from single trials or systematic reviews, but the link between the listed intervention, the references quoted and the magnitude of effect is not always clear. Prata [56] for example, lists two systematic reviews of the treatment of hypertension in support of a 48-65% reduction in eclampsia mortality, even though the review does not show evidence for an effect of hypertensive treatment on maternal death, eclampsia or pre-eclampsia. Similarly, Adam et al [57] report that antenatal screening for pre-eclampsia at the primary care level is 48% effective in the reduction of mortality from hypertensive diseases in pregnancy, relying on expert opinion and the systematic review showing no evidence for an effect of treating hypertension (Additional File 1). Bhutta et al, on the other hand, report that the treatment of hypertension reduces maternal death from HDP by 63%, without a clear reference to support this statement [60]. Bhutta et al also report a 30% reduction in mortality from HDP with routine calcium supplementation in pregnancy, referencing the review which reports a relative risk of 0.80 for the combined outcome of death and serious morbidity [60].

Five reviews estimate the reduction of a comprehensive package of hospital care for the management of pre-eclampsia and eclampsia [54,55,57][58-60] with estimates of effect ranging from no added benefit for caesarean sections [60] to a 76% reduction for a comprehensive package [55]. Even though a number of systematic reviews are quoted, it is not clear how these estimates are arrived at [57-59]. There are no randomised controlled trials for the combined medical treatment of eclampsia and pre-eclampsia, nor are there any trials quantifying the effect of a comprehensive package of obstetric care – including MgS04, labour induction and caesarean section – on mortality from hypertensive diseases.

### Comparing current and historic maternal mortality ratios across regions or countries

Table 1 shows HDP-specific maternal mortality by WHO region, and the risk reduction in mortality from HDP that can be expected if countries adopted current models of health care in developed countries. Using this approach, the risk reduction in mortality from HDP expected in Africa and Asia is 98.1% and 95.3% respectively.

**Table 1:** Effect of prevention and treatment of HDP on maternal mortality from HDP comparing hypertensive disease mortality in 2005 in developed countries with WHO region hypertensive disease mortality in 2005

	Africa (2005)	Asia (2005)	Latin America and the Caribbean (2005)	Developed countries (2005)
**Maternal deaths per 100,000 live births **[7,9]	820	330	130	9.0
**Hypertensive disease deaths per 100,000 live births (%)**	74.6 (9.1%)	30.0 (9.1%)	33.4 (25.7%)	1.4 (16.1%)
**Mortality reduction assuming developed country models in 2005 as optimal ^a^**	98.1%	95.3%	95.8.5%	NA

^a^ Mortality reduction was calculated as (Regional maternal mortality ratio (MMR) – MMR in developed countries)/ Regional MMR)

**Figure 1:**
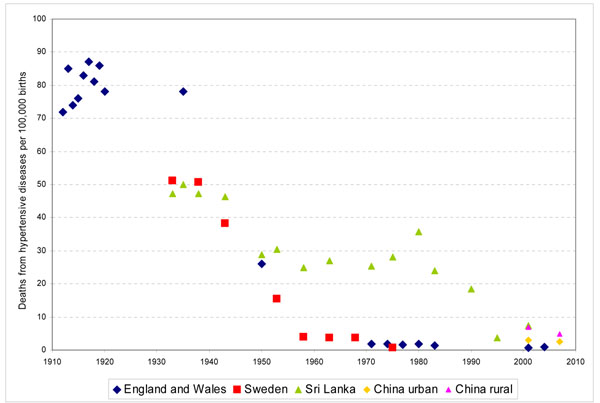
Trends in mortality from HDP in England & Wales, Sweden, Sri Lanka and urban and rural China.

Figure 1 shows trends in HDP-specific maternal mortality over time in England & Wales, Sweden, Sri Lanka and urban and rural China. Definitions of deaths from HDP are likely to vary between country and over time, though definitions are rarely provided. In the UK, the data reported are for puerperal convulsions between 1832 and 1876, toxaemia in 1950, and HDP (eclampsia and pre-eclampsia) for more recent years [10,11,66].[10,11,66] The trends in mortality from hypertensive diseases are remarkably similar in England & Wales and Sweden. It is interesting to note that both England & Wales achieved very low levels of mortality from hypertensive diseases in the 1970s, long before MgS04 was introduced. Urban and rural China and Sri Lanka achieved equally low levels by the 21^st^ Century.

Using the levels of mortality reported in England & Wales in 1912 as the starting point, England & Wales achieved a fall in mortality from hypertensive diseases of 98.8% while Sweden, Sri Lanka and urban and rural China achieved reductions of 99.0%, 89.7%, 96.5 and 93.3% respectively. Similarly, using Sweden’s reported mortality ratios for 1933 as a reference, Sweden achieved a mortality reduction of 98.6%, while England & Wales, Sri Lanka and urban and rural China achieved a reduction of 98.3%, 85.5%, 95.1 and 90.6% respectively.

### Comparing current and historic estimates of fatality in women with SAMM related to HDP

Table 2 shows case fatality rates in women with SAMM associated with HDP in high income countries. Case definitions vary, and a detailed discussion is provided elsewhere ^15^. The first study shows trends in fatality rates associated with eclampsia in Sweden from 1861 to 1980.[12] Between 1861 and 1888, at a time when effective treatment for HDP was unavailable, 42% of women were thought not to survive an episode of eclampsia. This proportion fell dramatically in the late 19^th^ Century when induction of labour was introduced, and by the late 20^th^ Century mortality in women with eclampsia was estimated to be as low as 3.1%. Using the Swedish estimates of fatality from eclampsia between 1861 and 1980, the risk reduction in mortality from eclampsia observed in Sweden in this period was 92.6%.

**Table 2:** Effect of treatment of HDP on maternal mortality from HDP using severe acute maternal morbidity (SAMM) case fatality rates in high income countries

Author, year	Study population (year)	HDP-related conditions	Case fatality rate (Number of deaths/cases)	Mortality reduction ^a^	
				**Natural case fatality**	
				**25%**	**42%**
**Hogberg 1985 **[12]	Sweden (1861-1880)	Eclampsia	42.0	-	-
	Sweden (1881-1900)	Eclampsia	20.0	20.0%	52.4%
	Sweden (1951-1955)	Eclampsia	13.9	44.4%	66.9%
	Sweden (1971-1980)	Eclampsia	3.1	87.6%	92.6%
**Douglas et al 1994 **[67]	UK (1992)	Eclampsia	1.8% (7/382)	92.7%	95.6%
**Waterstone et al 2001 **[68]	South East Thames Region (1997-1998)	Severe pre-eclampsia, eclampsia and HELLP syndrome	0.4% (1/224)	98.2%	98.9%
**Zhang et al 2005 **[69]	Upper Austria (1996-1997)	evere pre-eclampsia, eclampsia and HELLP syndrome	0.0% (0/32)	100.0%	100.0%
	Brussels, Belgium (1996)		0.0% (0/115)	100.0%	100.0%
	Finland, 65% of deliveries (1996)		0.0% (0/86)	100.0%	100.0%
	Four regions in France (1995)		0.4% (1/241)	98.3%	99.0%
	Upper Danube, Hungary (1995)		0.0% (0/81)	100.0%	100.0%
	Cork, Ireland (1996)		0.0% (0/9)	100.0%	100.0%
	Puglia, Italy (1996-1997)		0.0% (0/19)	100.0%	100.0%
	Oslo, Norway (1995)		0.0% (0/6)	100.0%	100.0%
**Wen et al 2005 **[70]	Canada (1991-1992)	Eclampsia	0.4% (4/973)	98.4%	99.0%
**Knight et al 2007 **[71]	UK (2005-2006)	Eclampsia	0.0% (0/214)	100.0%	100.0%
**Zwart et al 2008 **[72]	The Netherlands (2004-2006)	Eclampsia and HELLP syndrome	1.8% (4/222)	92.8%	95.7%

^a^ Mortality reduction was calculated as (Natural case fatality rate – SAMM fatality rate / Natural case fatality rate)

Case fatality rates in women with SAMM from HDP in contemporary high income countries are very low, ranging from no deaths in most regions to 1.8% in the UK in 1992 and the Netherlands between 2004 and 2006. This suggests an effectiveness of the current treatment of SAMM from HDP to be at least 93 to 96% (using 25% and 42% as natural case fatality rates respectively).

Table 3 shows case fatality rates in women with SAMM associated with HDP in low income countries. A detailed discussion of variation in case definitions is provided elsewhere [16]. Case fatality rates in women hospitalised with a SAMM from HDP range from 0.5% in hospitals in Bolivia to 20.8% in hospitals in South Africa. Using the “natural” case fatality rate in women with eclampsia reported for Sweden (42%) and England & Wales (25%), we report a minimum and maximum estimate of effectiveness of the existing treatment of severe cases of HDP in the various hospitals. Lower bound estimates range from 16.7% in hospitals in South Africa to 98.0% in hospitals in Bolivia. Similarly, higher bound estimates range from 50.4% in South Africa to 98.8% in Bolivia.

**Table 3:** Effect of treatment of HDP on maternal mortality from HDP using severe acute maternal morbidity case fatality rates in low income countries

Author, year	Study population (period)	Definition of HDP-related SAMM	Case fatality rate (Number of deaths/cases)	**Mortality reduction**^a^	
	
				Natural case fatality	
	
				25%	42%
**Mantel et al, 1998**[73]	Kalafong and Pretoria hospitals, South Africa (1996-1998)	Hypertension with organ failure	20.8% (10/48)	16.7%	50.4%
**Khosla et al 2000**[74]	One hospital, Rohtak, India (1998)	Eclampsia	17.7% (11/62)	29.0%	57.8%
**Pattinson et al, 2003**[75]	Three regions, South Africa (year not stated)	Hypertension with organ failure	20.7% (30/145)	17.2%	50.7%
**Kaye et al 2003**[76]	Mulago hospital, Uganda (2000)	Eclampsia/pre-eclampsia with organ failure	14.3% (3/21)	42.9%	66.0%
**Oladapo et al 2007**[77]	Olabisi Onabanjo hospital, southwest Nigeria (2002-2004)	Eclampsia and severe pre-eclampsia with clinical/laboratory indications for termination of pregnancy to save the woman's live	14.9% (21/141)	40.4%	64.5%
**Adisasmita et al 2008**[78]	Four hospitals in West-Java, Indonesia (2003-2004)	Eclampsia and pre-eclampsia with organ failure	3.9% (22/563)	84.4%	90.7%
**Roost et al 2009**[79]	Four public hospitals in La Paz and El Alto, Bolivia (2006-2007)	Eclampsia and pre-eclampsia based on clinical and management criteria	0.5% (1/184)	98.0%	98.8%

^a^ Mortality reduction was calculated as (Natural case fatality rate – SAMM fatality rate / Natural case fatality rate)

### Intervention effects for the Lives Saved Tool (LiST) model

Our recommended estimates for the LiST model are shown in Table 4. Where evidence from systematic reviews was available we used the judgement rules proposed by Walker et al [5]. Where no such evidence was available we relied on our estimates of effect from comparisons of case fatality rates in SAMM from HDP in countries with the lowest case fatality rates (Indonesia and Bolivia) and from historical trends in HDP mortality in China, Sri Lanka, Sweden and England and Wales.

**Table 4:** Potential effect of prevention and treatment of HDP on maternal mortality from HDP using evidence from systematic reviews, historical trends and fatality rates in SAMM from HDP

Health system level at which intervention is delivered	Intervention	Risk reduction on HDP-related mortality	Source of effectiveness estimate
Health centre	Calcium supplementation during pregnancy	20%	Systematic review of effect of routine calcium supplementation versus placebo on death/serious morbidity[40]
(L)District or secondary hospital	Calcium supplementation during pregnancy	20%	Systematic review of effect of routine calcium supplementation versus placebo on death/serious morbidity[40]
	MgS04 for pre-eclampsia	59%	Systematic review of effect of MgS04 versus placebo for treatment of pre-eclampsia on eclampsia[45]
	MgS04 for eclampsia	41%	Systematic review of effect of MgS04 versus diazepam for treatment of eclampsia on death[42]
	Antenatal screening for hypertension and proteinuria and treatment of pre-eclampsia and eclampsia with MgS04 and early delivery in women with severe pre-eclampsia and eclampsia	84-99%	Case fatality rates in SAMM from HDP in Indonesia[78] and Bolivia[79] and historical trends in HDP mortality in China and Sri Lanka
Tertiary hospital	All the above plus treatment of severe hypertension in pregnancy and referral to specialist intensive care for women with severe complications	99%	Historical trends in HDP mortality in Sweden and England & Wales

## Discussion

This study highlights the complexity in quantifying the effect of interventions to prevent or treat HDP on mortality from HDP. First, evidence based on the accepted scientific standard - randomised controlled trials - is only available for a small number of single interventions, and no randomised trial evidence is available for interventions which have been highly effective in reducing HDP-related mortality in high income countries, such as caesarean sections. Second, only one intervention (MgS04 for eclampsia) has been shown to have an effect on mortality. Third, the magnitude of effect on severe outcomes is not always consistent with that seen in less severe outcomes (e.g. Calcium supplementation reduces death/serious morbidity by 20% and pre-eclampsia by 52%). Fourth, estimates of effectiveness of comprehensive intervention packages – whether extrapolated from trends in HDP mortality over time or between regions, or from case fatality rates from SAMM due to HDP in high and low income countries – vary greatly, and interpretation is hampered by lack of knowledge on the content of these intervention packages. Existing models have tried to circumvent these problems by seeking advice from “experts” and/or by narrowly focusing on the efficacy of single interventions, but the inconsistencies, ambiguity and lack of transparency in the assumptions underlying the models call into question the validity of reported effect estimates.

The 98% fall in HDP related mortality in the UK and Sweden over fifty years suggests that HDP-related deaths are highly avoidable. The fall in mortality from HDP has been largely attributed to a reduction in the number of cases of eclampsia, while the incidence of pre-eclampsia has been more resistant to change [10,67]. The package of interventions that has caused this drop is not known with certainty, but antenatal screening for high blood pressure and proteinuria in the second half of pregnancy, with early delivery through induction of labour or caesarean delivery in women diagnosed with pre-eclampsia is thought to be the main reason [10]. Induction of labour after 36 weeks gestation in women with gestational hypertension or mild pre-eclampsia is clearly effective in reducing adverse maternal outcomes [39]. Optimal timing of delivery before 32-34 weeks in women with pre-eclampsia remains a dilemma because of the uncertain balance between protecting the mother (by ending pregnancy) and enhancing the maturity of the baby (by delaying delivery) [30,80]. After 34 weeks gestation, however, the survival of babies in high income countries is nearly 100% and ending the pregnancy by delivering the baby and the placenta is highly effective [10]. The routine introduction of MgS04 for the treatment of pre-eclampsia and eclampsia in more recent years is likely to have further reduced mortality, explaining the very low case fatality rates seen in women with SAMM from HDP in high income countries today.

Can these findings be applied to low income countries? The low levels of HDP related mortality in rural China and Sri Lanka suggest that reductions of 85% or more are within reach, provided that most women give birth with a health professional who can refer them to higher levels of care when necessary. Results from SAMM studies in Indonesia and Bolivia also suggest that mortality in women with severe pre-eclampsia or eclampsia in hospital can be reduced by more than 84%, even when the women arrive late [78,79]. The high variability in case fatalities for SAMM from HDP does not necessarily call into question the potential effectiveness of a tertiary prevention strategy for HDP. It reflects the reality of poor quality of care in some settings, including limited availability of essential drugs, poor adherence to standard guidelines, and huge delays in receiving emergency care [81]. The availability and use of MgS04 remains poor, even though the drug is cheap and has appeared on the WHO’s essential medicines list since 1996 [82]. Secondary prevention of HDP, with screening for hypertension and proteinuria should also be possible, but organising referral care once problems occur remains a huge challenge [83].

Various authors have attempted to identify interventions that can be delivered at health centre level in the absence of referral care, but the range of interventions varies greatly. Dietary supplementation with at least 1 g of calcium a day reduces the risk of death or serious morbidity from HDP by 20%, and the effect may be stronger for high risk women or those with low dietary calcium. Calcium is cheap and it should be possible to achieve high coverage antenatally through a health centre delivery strategy, though the intervention should be started early in pregnancy. Other interventions are not likely to work at the health centre level alone. Low dose aspirin reduces the risk of pre-eclampsia by 17%, but there is no evidence of an effect on eclampsia or death. Antihypertensive drugs in mild to moderate hypertension during pregnancy halve the risk of severe hypertension, but they do not prevent or delay progression to pre-eclampsia or eclampsia.[80] Treatment of severe hypertension is essential, but the choice of drug is not obvious, and an experienced clinician needs to decide on a case by case basis [46]. MgS04 has been suggested for use at the primary care level, but MgS04 is difficult to administer, and health centres need to refer women to hospital, even if they are able to give a loading those.

Table 4 suggests potential effect estimates for the reduction of HDP related mortality based on this review. We postulate that health centres without access to referral care can contribute to a 20% reduction in death from HDP through supplementation of pregnant women with calcium, but there is little scope for further reductions unless referral care is available. If women have access to a hospital with qualified staff and drugs, much greater reductions can be expected. A package of interventions including the detection of hypertension and proteinuria in pregnancy and treatment of pre-eclampsia and eclampsia with MgS04 and early delivery can probably reduce HDP mortality by at least 84%, as was seen in Indonesia and China. Further reductions require treatment for severe hypertension and referral to specialist intensive care for women with severe complications, as is offered in the UK [67].

We have based our estimates of the effect of packages of interventions on observational data, comparing current HDP mortality or case fatality with that at times when no effective interventions were available. This approach has a number of limitations. First, we assume that variation in the incidence of HDP mortality or case fatality is entirely due to health service factors, while there is no biological or genetic variation between populations. This is clearly not true. The incidence of HDP varies by parity and maternal age, and variation in the demographic profile of the population will affect the incidence and mortality from HDP. Second, maternal mortality and SAMM are difficult to measure [2,15,16] and variations in case definitions may have affected the results. Eclampsia may be one of the more easily recognised clinical entities, however, and errors less marked than for other obstetric problems. Third, unlike SAMM from other diseases which have a case fatality rate close to 100% if untreated, women can survive eclampsia in the absence of treatment. We used historical data from the UK and Sweden to estimate the natural case fatality rate but such data are clearly imprecise, and results have to be interpreted with caution.

The increasing emphasis on the rating of the quality of evidence has led to greater reliance on evidence from randomised controlled trials to estimate the effect of interventions. Yet evidence from randomised studies is often not available, the effects observed on morbidity may not translate in to mortality, and the distinction between efficacy and effectiveness may be difficult to make. We suggest that more use should be made of observational evidence, particularly since such data represent the actual effectiveness of packages of interventions in various settings.

## Competing interests

The authors declare no conflict of interest.

## Supplementary Material

Additional file 1 .xlsx. Efficacy of treatment and prevention of HDP based on Cochrane reviews. Click here for file

Additional file 2 .xlsx. Review of population models quantifying the effect of prevention and treatment of HDP on maternal mortality from HDP. Click here for file
